# Comparative transcriptomic analysis-based identification of the regulation of foreign proteins with different stabilities expressed in *Pichia pastoris*

**DOI:** 10.3389/fmicb.2022.1074398

**Published:** 2022-12-22

**Authors:** Tingting Niu, Yi Cui, Xu Shan, Shuzhen Qin, Xuejie Zhou, Rui Wang, Alan Chang, Nan Ma, Jingjing Jing, Jianwei He

**Affiliations:** ^1^School of Life Sciences, Liaoning University, Shenyang, China; ^2^College of Life and Environmental Sciences, Wenzhou University, Wenzhou, China; ^3^China Academy of Transportation Sciences, Beijing, China; ^4^Tumor Etiology and Screening Department of Cancer Institute and General Surgery, The First Hospital of China Medical University, Shenyang, China

**Keywords:** cystatin, transcriptomic, amyloid disease, *pichia pastoris*, biofuel

## Abstract

**Introduction:**

The industrial yeast *Pichia pastoris* is widely used as a cell factory to produce proteins, chemicals and advanced biofuels. We have previously constructed *P. pastoris* strains that overexpress protein disulfide isomerase (PDI), which is a kind of molecular chaperone that can improve the expression of an exogenous protein when they are co-expressed. Chicken cystatin (cC) is a highly thermostable cysteine protease inhibitor and a homologous protein of human cystatin C (HCC). Wild-type cC and the two mutants, I66Q and ΔW (a truncated cC lacking the á-helix 2) represent proteins with different degrees of stability.

**Methods:**

Wild-type cC, I66Q and ΔW were each overexpressed in *P. pastoris* without and with the coexpression of PDI and their extracellular levels were determined and compared. Transcriptomic profiling was performed to compare the changes in the main signaling pathways and cell components (other than endoplasmic reticulum quality control system represented by molecular chaperones) in *P. pastoris* in response to intracellular folding stress caused by the expression of exogenous proteins with different stabilities. Finally, hub genes hunting was also performed.

**Results and discussion:**

The coexpression of PDI was able to increase the extracellular levels of both wild-type cC and the two mutants, indicating that overexpression of PDI could prevent the misfolding of unstable proteins or promote the degradation of the misfolded proteins to some extent. For *P. pastoris* cells that expressed the I66Q or ΔW mutant, GO (Gene Ontology) and KEGG (Kyoto Encyclopedia of Genes and Genomes) analyses of the common DEGs in these cells revealed a significant upregulation of the genes involved in protein processing, but a significant downregulation of the genes enriched in the Ribosome, TCA and Glycolysis/Gluconeogenesis pathways. Hub genes hunting indicated that the most downregulated ribosome protein, C4QXU7 in this case, might be an important target protein that could be manipulated to increase the expression of foreign proteins, especially proteins with a certain degree of instability.

**Conclusion:**

These findings should shed new light on our understanding of the regulatory mechanism in yeast cells that responds to intracellular folding stress, providing valuable information for the development of a convenient platform that could improve the efficiency of heterologous protein expression in *P. pastoris.*

## 1 Introduction

Human cystatin C (HCC) is a papain-like cysteine protease inhibitor that belongs to the cystatin superfamily, and it is also one of the most extensively studied endogenous inhibitors as well as an important biomarker of renal function (Dubin, 2005). Abnormal changes in the expression and secretion of HCC in the brain have been described for various neurological disorders such as amyotrophic lateral sclerosis (ALS), rare heritable neurodegenerative disorders, ischemia, some forms of epilepsy, Alzheimer’s disease (AD) (Mathews and Levy, 2016) and recurrent hemorrhagic stroke (Merz et al., 1997; Zhou et al., 2015).

Previous studies have reported that the fatal amyloid disease, hereditary cystatin amyloid angiopathy (HCCAA), found in young Icelanders is mainly caused by the HCC hereditary amyloidogenic mutant L68Q, which has a high dimerization potential that can lead to self-aggregation and hyper-amyloidosis (Janowski et al., 2001; Palsdottir et al., 2006). The instability of the soluble HCC monomer has constrained any structural studies on its physicochemical properties. Meanwhile, chicken cystatin (cC) has a number of characteristics similar to HCC, and both proteins share about 44% sequence homology. Thus, cC is considered an ideal model for studying protein domain exchange and amyloid-related diseases (Bode et al., 1988; Yu et al., 2010). Residue 66 in cC corresponds to residue 68 in HCC, and the I66Q mutant of cC has similar amyloidogenic properties to L68Q of HCC under physiological conditions (Bjarnadottir et al., 2001).

The AS (appending structure) region of cC contains α-helix 2, which is crucial for the stability of cC and is considered to be the biggest difference between HCC and cC (Grubb et al., 1984). Therefore, the α-helix 2-truncated mutant (ΔW) with a deletion at residues 77–85 was constructed as the unstable cC model protein. Based on our previous results, the secreted amount of ΔW is much lower than that of WT cC or I66Q when expressed in *P. pastoris*, indicating that the absence of α-helix 2 in the AS region may be one of the factors contributing to the structural instability of HCC (Zhou et al., 2019).

**FIGURE 1:**
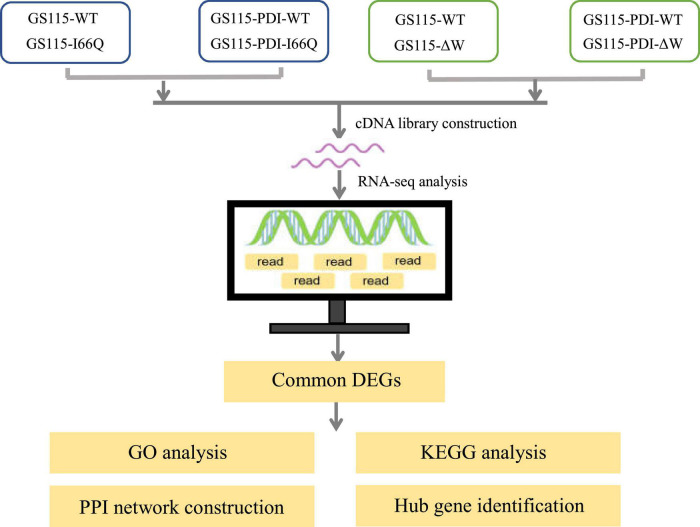
Schematic diagram of the workflow conducted in this study.

*Pichia pastoris* (reclassified as *Komagataella phaffii/pastoris*) is a methylotrophic yeast and a highly successful system for producing recombinant proteins in the pharmaceutical and biofuel industries (Yu et al., 2017). The ability of *P. pastoris* to express recombinant proteins is facilitated by the strong promoter of its alcohol oxidase 1 (AOX1) gene. The activity of the AOX1 promoter is tightly regulated by the carbon source. Thus, recombinant proteins expressed from the AOX1 promoter in *P. pastoris* cells can be induced with methanol once cell growth has reached high densities to obtain a high level of expression for the proteins. For example, a His-Qtagged lipase A from *Beauveria bassiana* has been successfully produced in *P. pastoris* and shown to have potential use for biodiesel production *via* ethanolysis (Vici et al., 2015). Another example is the expression of α-L-arabinofuranosidase (ARA) in *P. pastoris*, which can be improved 5.5-fold by codon optimization. The recombinant ARA has significant potential in the catalytic conversion of corn stover to fermentable sugars during biofuel production (Vici et al., 2015). However, overexpression of recombinant proteins may lead to more misfolded proteins and trigger endoplasmic reticulum (ER) stress (Jolly and Morimoto, 2000; Oakes and Papa, 2015). The cells might then respond to ER stress by increasing the expression of some molecular chaperones, including PDI, HSP90, and HSP72 (Vogl and Glieder, 2013; Delic et al., 2014; Gu et al., 2015). To prevent protein misfolding and aggregation, the newly synthesized molecular chaperones would increase folding efficiency by capturing the folded intermediates and promoting refolding or degradation. Co-expression of PDI has been used to improve the expression of heterologous proteins in *P. pastoris* by overcoming the burden of protein folding and secretion (Inan et al., 2006; Navone et al., 2021a). In our previous study, the overexpression of PDI in *P. pastoris* GS115 strains was found to significantly increase the expression of cC (Zhou et al., 2019). On this basis, three representative proteins with different stabilities (WT cC and its two mutants I66Q and ΔW) (Figure 1) were used as model proteins to screen for factors other than the ER quality control system represented by the molecular chaperone PDI that could influence the expression of foreign proteins that may not be properly folded in *P. pastoris*. Subsequently, transcriptomic profiling was performed to identify the transcriptomic changes and pathways involved in the molecular network and changes in the dynamic mechanism of the foreign protein secretion pathway in *P. pastoris*.

## 2 Materials and methods

### 2.1 Strains, plasmids, and culture conditions

*Pichia Pastoris* GS115 strain was provided by Dr. Shutao Liu at Fuzhou University. The plasmids pPIC3.5K and pPICZαA were purchased from Invitrogen. GS115 strain and the previously constructed GS115 PDI-overexpressing strain were used as starting strains for the construction of the PDI and cC co-overexpressing strains. The yeast cells were first cultured at 30^°^C in Yeast Peptone Dextrose medium (YPD) (1% yeast extract, 2% peptone, and 2% glucose) to logarithmic growth phase (OD_600_ = 5.0) followed by methanol induction in Yeast Extract Peptone Medium (YPM) [1% yeast extract, 2% peptone, 0.5% Methanol (v/v)] for 72 h to induce the expression of PDI and cC.

### 2.2 Construction of recombinant strains

GS115 competent cells were transformed with the linearized plasmid pPICZαA-cC, pPICZαA-I66Q, and pPICZαA-ΔW by electroporation to generate GS115-cC, GS115-I66Q, GS115-ΔW recombinant strains, respectively. Similarly, GS115-PDI-cC, GS115-PDI-I66Q, GS115-PDI-ΔW strains were obtained by transforming GS115 PDI with pPICZαA-cC, pPICZαA-I66Q, and pPICZαA-ΔW, respectively.

### 2.3 Protein expression analysis

Extracellular protein samples were obtained as previously described (Zhou et al., 2019). Intracellular protein samples were extracted from yeast cells after treatment with Yeast Protein Extraction Reagent (Takara, Dalian, China). SDS-PAGE and western blotting were carried out following the procedure described previously (Zhou et al., 2019). The protein bands in one gel were visualized by staining the gel with PAGE Gel Silver Staining Kit (Takara, Beijing, China) whereas the protein bands in the other gel were transferred to a PVDF membrane (Millipore, MA, USA) for western blot analysis. After protein transfer, the PVDF membrane was incubated in a blocking buffer containing TBST plus 5% skimmed milk powder for 2 h. This was followed by three 10 min washes in TBST buffer, and 1 h of incubation in rabbit anti-cC antiserum (1:2000) at room temperature. After that, the membrane was again washed three times in TBST, with each wash lasting for 10 min. Finally, the blot was incubated with anti-rabbit peroxidase conjugate (1:10000) for 1 h at RT, and then subjected to detection using the eECL Reagent (Beyotime, Shanghai, China).

### 2.4 RNA sequencing

Total RNA was extracted from the different GS115 strains using Yeast RNAiso Kit (Takara, Dalian, China). The extraction was performed according to the manufacturer’s protocol. The mRNA fraction was purified from the total RNA using MicroPoly Purist kit (Takara, Dalian, China) according to the manufacturer’s protocol. The concentration and integrity of the mRNA were measured using a NanoDrop 2000 (Thermo Fisher Scientific, MA, USA) and the Agilent 2100 LabChip system (Agilent Technologies, CA, USA). The RNA was sheared, and reverse transcribed using random primers to obtain the cDNA, which was then used for the construction of a cDNA library. Illumina RNA sequencing (RNA-Seq) libraries were subsequently performed using VAHTS Universal V6 RNA-seq Library P the SMARTer Stranded RNA Seq Kit (Vazyme Biotech Co. Ltd.) according to the manufacturer’s instructions. Finally, RNA-Seq data were generated in Fastq format. The sequencing data have been submitted to National Center for Biotechnology Information (NCBI) under accession PRJNA892887^1^.

### 2.5 Screening for differentially expressed genes

The differentially expressed genes (DEGs) for WT vs. I66Q, PDI-WT vs. PDI-I66Q, WT vs. ΔW and PDI-WT vs. PDI-ΔW were identified by the BioMarker cloud platform with adjusted *p*-value <0.01 and log_2_ fold change (FC) >2. Moreover, the common DEGs between different groups have been identified by the same method.

### 2.6 Functional enrichment analyses for common DEGs

Gene Ontology (GO) and Kyoto Encyclopedia of Genes and Genomes (KEGG) analyses were performed on the BioMarker cloud platform with a *p*-value <0.05. ClueGo plug-in in Cytoscape software (3.8 version) was used for showing the ClueGO network diagram.

### 2.7 Protein–protein interaction network construction and hub gene identification

Common DEGs were used to construct the protein–protein interaction (PPI) network by using the SRTING online database with a confidence score of more than 0.7. Hub genes of the PPI network were identified using a degree algorithm from cytoHubba, a plugin in Cytoscape, and visualized using Cytoscape (v3.8.0).

## 3 Results

### 3.1 Effect of PDI-overexpression on the expression of WT cC and cC mutants

**FIGURE 2:**
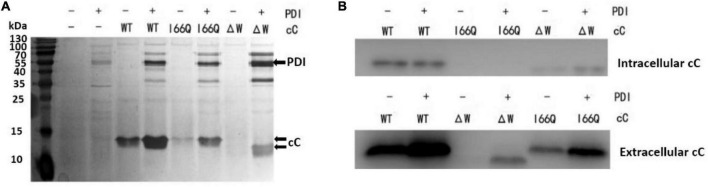
Analysis of the expression of the different versions of chicken cystatin (cC) (MW: 13 kDa) in both GS115 strains that did and did not overexpress PDI (MW: 57 kDa). **(A)** Secretion of the WT cC, I66Q and ΔW as detected in the culture supernatant after centrifugation as shown by SDS-PAGE. The gel was visualized by silver staining. **(B)** Expression of WT cC, I66Q and ΔW in GS115 as analyzed by western blot. Intracellular refers to the soluble fraction of cell lysate, and Extracellular refers to the culture supernatant. “+” and “–” indicates the cells were transfected with and without the corresponding cC-coding gene, respectively.

To investigate the intracellular distribution pattern and retention level of recombinant cC as well as its extracellular secretion in *P. pastoris*, WT cC, I66Q and ΔW were expressed in both *P. pastoris* GS115 strains without and with the overexpression of PDI. The extracellular secretion of wild-type cC and its two mutants was detected by silver staining (Figure 2A). Overexpression of PDI (57 kDa) can significantly enhance the expression of WT cC (14 kDa) and the I66Q mutant. In the case of ΔW, the protein was only secreted when it was co-expressed with PDI (Figure 2B). As shown in Figure 2B, almost no I66Q and ΔW were detected as an intracellular form in both PDI-overexpressing GS115 and wild-type GS115 strains. Interestingly, for WT cC, no significant difference in intracellular level was observed between the two yeast strains. This might suggest that when the yeast expressed WT cC, most of the proteins were capable of folding into the native form and were subsequently secreted out of the cell, with little misfolded protein being produced and residing in the ER despite an increase in the amount of the newly synthesized protein entering the ER. For the mutant I66Q, its intracellular level was proportional to its extracellular level in either yeast strain because of its amyloidogenic properties. Consequently, the three cC-overexpressing GS115 strains and the three cC-overexpressing GS115 strains that also co-expressed PDI were used for the following transcriptomic studies.

### 3.2 Transcriptomic analysis of different recombinant GS115 strains

**FIGURE 3:**
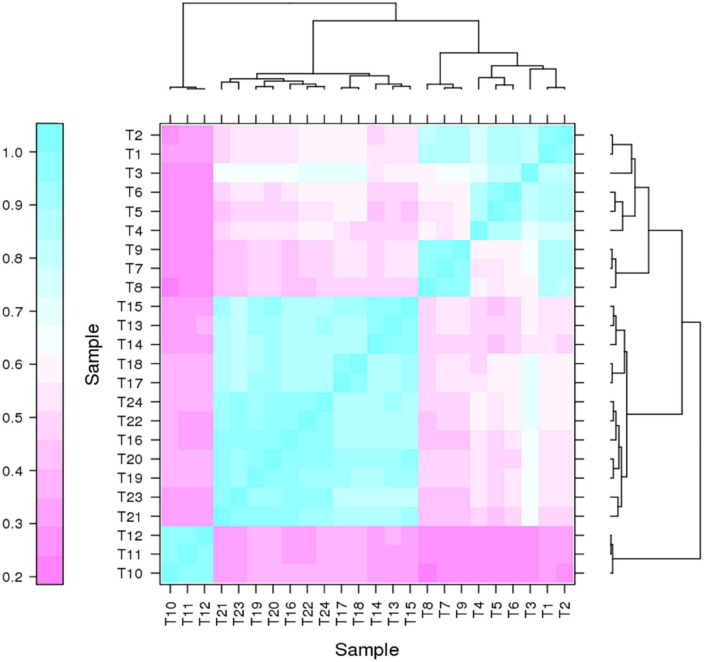
Correlation heat map for two pairs of samples.

It has been proven that genes are being expressed at different levels in different individual organisms as a result of biological variability (Robasky et al., 2014). Therefore, biological replicates were included to ensure the validity of the following experiments. The Pearson Correlation Coefficient r refers to the biological assessment of repeated samples and it was used to analyze the correlation between every two samples (Schulze et al., 2012). The closer r^2^ is to 1, the stronger the correlation between the two replicates (Figure 3). Subsequently, 24 samples were subjected to the transcriptomic analysis after RNA-sequencing was completed, with three replicates included for each strain and the data are summarized in Table 1. The clean reads of each sample were compared with the designated *P. pastoris* GS115 genome. The mapped data obtained after alignment were used to evaluate the quality of the library such as randcheck, insert size, and saturation test. Typically, FPKM (Fragments Per Kilobase of transcript per Million fragments mapped) was used as an indicator to measure the expression level of a gene (Zhao et al., 2021). Identification of DEGs was carried out according to the gene expression levels in different samples.

**TABLE 1:** Summary of the samples analyzed by RNA—sequencing (RNA-seq).

Samples for RNA-seq	Strain	Overexpressed protein
T1, T2, T3	GS115	/
T4, T5, T6	GS115-PDI	PDI
T7, T8, T9	GS115-cC	cC
T10, T11, T12	GS115-PDI-cC	PDI/cC
T13, T14, T15	GS115-I66Q	I66Q
T16, T17, T18	GS115-PDI-I66Q	PDI/I66Q
T19, T20, T21	GS115-ΔW	ΔW
T22, T23, T24	GS115-PDI-ΔW	PDI/ΔW

**FIGURE 4:**
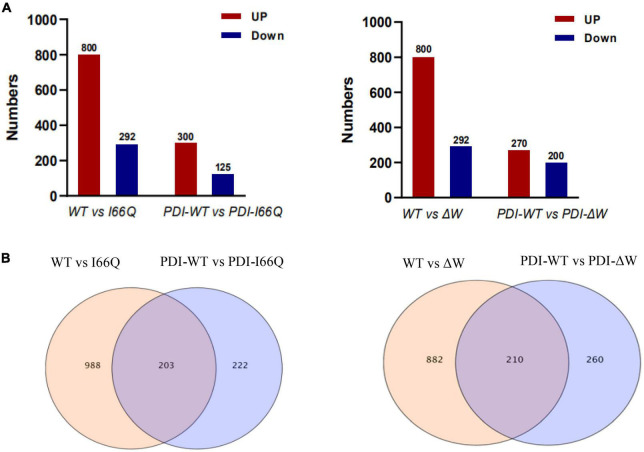
Count of differentially expressed genes (DEGs) in WT vs. I66Q/ΔW and PDI-WT vs. PDI-I66Q/ΔW comparisons. **(A)** Total number of up and downregulated DEGs in the four pairs: WT vs. I66Q, WT vs. ΔW, PDI-WT vs. PDI-I66Q and PDI-WT vs. PDI-ΔW. **(B)** Venn diagram depicting the total number of proteins, including the up and down-regulated genes in the four groups.

Different numbers of up-and down-regulated DEGs were obtained by comparing each of the two sample groups as shown in Figure 4. Interestingly, when the WT cC-overexpressing strain was compared with either the I66Q- or ΔW-overexpressing strain, the DEGs, especially the up-regulated genes, were significantly increased. Nevertheless, this trend was not observed in the comparison of PDI-WT vs. PDI-I66Q/ΔW, implying a healthy intracellular cell condition in GS115-PDI-I66Q and GS115-PDI-ΔW afforded by the overexpression of PDI. From this point, it became important to investigate the common DEGs identified from the comparison of both WT vs. I66Q/ΔW and PDI-WT vs. PDI-I66Q/ΔW, since these common DEGs are vital for the overexpression of foreign proteins that are less stable. The total number of up-regulated and down-regulated genes in each comparison group was depicted in a Venn diagram (Figure 4B). Among all the compared genes, 203 and 210 common DEGs indicated in Figure 4B were selected for subsequent KEGG analysis, GO annotation, and hub gene identification.

### 3.3 KEGG analysis of common DEGs

**FIGURE 5:**
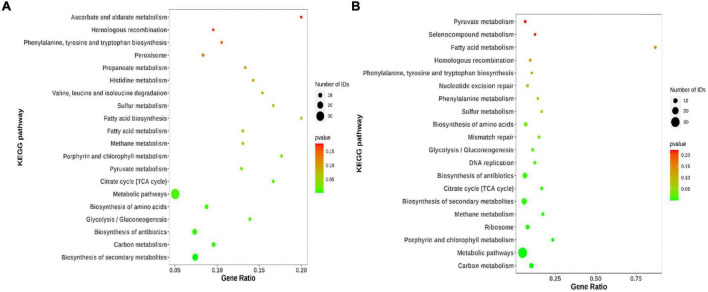
Kyoto encyclopedia of genes and genomes (KEGG) pathway enrichment analysis of common differentially expressed genes (DEGs). **(A)** KEGG pathway enrichment of 203 common DEGs identified from the WT vs. I66Q and PDI-WT vs. PDI-I66Q comparisons. **(B)** KEGG pathway enrichment of 210 common DEGs from the WT vs. ΔW and PDI-WT vs. PDI-ΔW comparisons.

Kyoto encyclopedia of genes and genomes analysis was applied to explore the potential molecular functions and molecular mechanisms associated with the functions of the common DEGs. As shown in Figure 5A, for WT vs. I66Q and PDI-WT vs. PDI-I66Q comparisons, several signaling pathways were significantly enriched, including the pathways for the biosynthesis of amino acids, citrate cycle, and Tricarboxylic Acid cycle (TCA cycle), and the metabolic pathways. In the comparison of WT vs. ΔW and of PDI-WT vs. PDI-ΔW, in addition to the pathways that were enriched in both WT vs. I66Q and PDI-WT vs. PDI-I66Q, the DNA replication and ribosome pathways were also indicated, suggesting that the activation of genes in different KEGG pathways may be due to the different physicochemical properties of the I66Q and ΔW cC mutants (Figure 5B).

**FIGURE 6:**
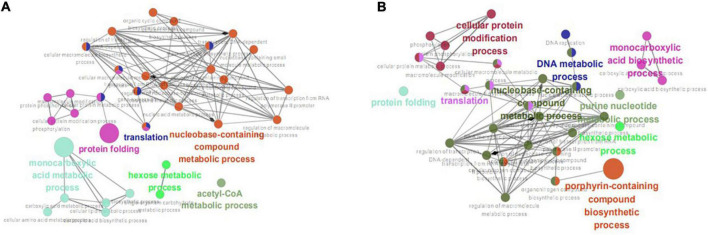
ClueGO enrichment analysis. **(A)** Significantly enriched gene ontology (GO) terms of the common differentially expressed genes (DEGs) identified from the WT vs. I66Q and PDI-WT vs. PDI-I66Q comparisons. **(B)** Significantly enriched GO terms of the common DEGs from the WT vs. ΔW and PDI-WT vs. PDI-ΔW comparisons.

Enrichment analysis was performed on the 203 and 210 common DEGs using the ClueGO v2.5.4 plugin. After setting the *P-*value as <0.05 and the Kappa Score Threshold as 0.4, 25, and 24 GO terms were identified from the group WT vs. I66Q and PDI-WT vs. PDI-I66Q, WT vs. ΔW and PDI-WT vs. PDI-ΔW, respectively. For the comparison of WT vs. I66Q and PDI-WT vs. PDI-I66Q, the enriched genes were mainly involved in protein folding, translation, and the acetyl-CoA metabolic and monocarboxylic acid biosynthetic processes (Figure 6A). For WT vs. ΔW and PDI-WT vs. PDI-ΔW, the enriched genes were not only distributed in the above pathways but also in the DNA metabolic process and porphyrin-containing compound biosynthetic process (Figure 6B). The result obtained from ClueGO enrichment analysis provided a global understanding of the scenario when proteins with different stabilities were expressed in *P. pastoris*. The common DEGs enriched in both the metabolic and protein processing pathways were quite noticeable in the comparison of WT vs. I66Q and that of WT vs. ΔW, suggesting that when amyloid mutants and unstable exogenous proteins are expressed in the *P. pastoris*, there might be a need to adjust the basic metabolic speed/efficiency and reproduction speed of the cells.

### 3.4 PPI network construction and hub genes identification

**FIGURE 7:**
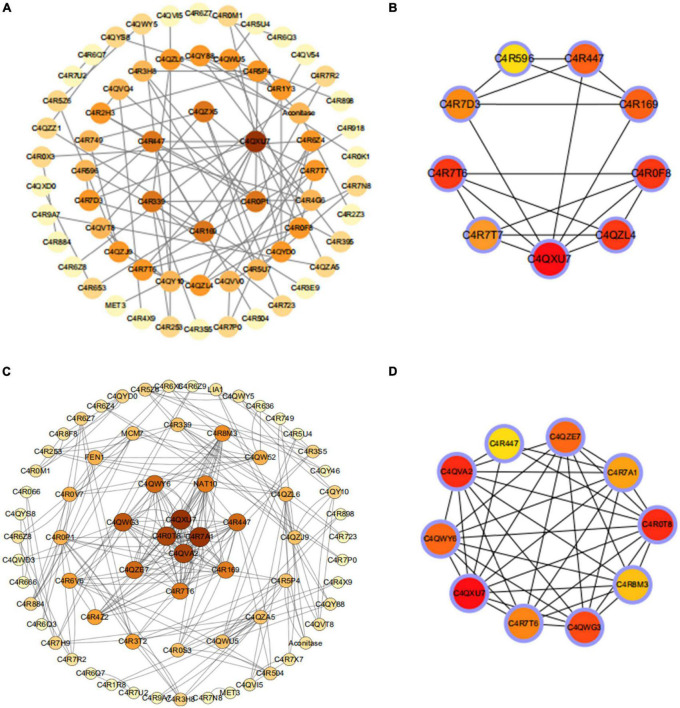
Protein–protein interaction (PPI) network of the proteins encoded by the common differentially expressed genes (DEGs) and selection of hub genes. **(A,B)** PPI network and the top 10 common DEGs between WT and I66Q and between PDI-WT and PDI-I66Q. **(C,D)** PPI network and the top 10 common DEGs between the group WT and ΔW and between PDI-WT and PDI-ΔW.

To further investigate the key cellular components and biological processes in the wild-type GS115 (I66Q/ΔW) and PDI-overexpressing GS115 strains (I66Q/ΔW), both of which were found to have common enriched DEGs, the STRING (Search Tool for the Retrieval of Interacting Genes) online tool was used to construct a PPI network of common DEGs. A combined score of >0.7 was set as the cut-off criterion for statistical significance. Next, the PPI network was downloaded and visualized as shown in Figure 7. A total of 132 nodes and 101 edges were identified for the common genes belonging to the comparison of WT vs. I66Q and of PDI-WT vs. PDI-I66Q (Figure 7A). According to the Degree sores in the cytoHubba, the top ten highest-scored genes were selected as the hub genes, including the common DEGs that encode the proteins C4QXU7, C4QZL4, C4R7T6, C4R447, C4R196, C4R0F8, C4R7T7, C4R7D3, C4R596 (Figure 7B). As for the comparison of WT vs. ΔW and of PDI-WT vs. PDI-ΔW, 140 nodes and 118 edges were found in the PPI network (Figure 7C), and the Hub genes included the common DEGs that encode C4QXU7, C4QVA2, C4R0T8, C4R447, C4QZE7, C4R7A1, C4R8M3, C4QWG3, C4R7T6, C4R7T6, C4QWY6 (Figure 7D).

C4QXU7, C4R7T6, and C4R0F8 were the top three key proteins identified in the comparison of WT vs. I66Q and of PDI-WT vs. PDI-I66Q. C4QXU7 is a small subunit of ribosomal protein S28e, whereas C4R7T6 is the large subunit of the ribosomal protein LP1, and C4R0F8 is a small subunit of the ribosomal protein S26e. In the case of WT vs. ΔW and PDI-WT vs. PDI-ΔW comparisons, the top three key proteins included C4QXU7, C4QVA2, and C4R0T8. C4QVA2 is a small subunit of ribosomal protein S3e, and C4R0T8 is a small subunit of ribosomal protein S15A. All of the above-mentioned proteins are responsible for the structural integrity of the ribosome and the protein translation rate. Interestingly, all the top three hub genes belong to the components of ribosomal subunits and they were found to be downregulated. Moreover, they were all found to be enriched in the translation pathway. This observation underlined the importance of the rate of protein synthesis by ribosomes and suggested that protein synthesis by ribosomes may be the ultimate goal that yeast cells need to adjust when an exogenous unstable protein is expressed in *P. pastoris.*

## 4 Discussion

It is generally believed that the amount of secreted amyloid mutant proteins and unstable mutant proteins is usually lower than that of their wild-type counterparts when eukaryotic expression systems are used (Whyteside et al., 2011; Bou Ali et al., 2013; He et al., 2019). When yeast cells express foreign proteins, the misfolded proteins may become toxic to the cells, and therefore, the co-overexpression of molecular chaperones may promote the folding and posttranslational modifications of the expressed proteins, thereby facilitating the secretion of the foreign proteins. Our results showed that co-expression of PDI with wild-type cC or mutated cC (I66Q or ΔW) could improve the secretion of cC to different extents. However, the distinct client-recruiting system of molecular chaperones may have a limit in improving the secretion of foreign proteins that may not be properly folded (Yan et al., 2021). This limitation could prevent *P. pastoris* from becoming an ideal protein production platform that can accommodate a variety of production requirements. Although modifications of specific transcription factors have been adopted to alter the regulation mode, it is still difficult to markedly increase the expression of foreign proteins in wild-type *P. pastoris* (Nusse and Lindau, 1988; Wang et al., 2017; Vogl et al., 2018). In this context, it is essential to pursue other regulatory genes and related cellular pathways that are capable of increasing the expression of foreign proteins in *P. pastoris*. Accordingly, WT, I66Q and ΔW were designed to be secreted by *P. pastoris* cells that did not overexpress PDI and those that did overexpress PDI, since both cell types could achieve different levels of protein expression in response to the various stabilities of WT cC and the two cC mutants. Our results, along with previously reported results, have shown that following their expression in *P. pastoris*, improperly folded foreign proteins may be found in lower levels compared with those that are properly folded, such as the improperly folded cC mutants versus their wide-type counterpart. Interestingly, the two cC mutants had different stabilities, which directly impacted their extracellular and intracellular levels. Consequently, the order of decreasing stability for WT cC, I66Q and ΔW turned out to be well-suited for establishing *P. pastoris* strains that could be used to investigate the key regulatory genes that the host cell would modulate when expressing foreign proteins that may not be properly folded.

Both *P. pastoris* GS115 which did not overexpress PDI and *P. pastoris* GS115 overexpressed PDI were used as the starting strains to express different variants of cC having different stabilities (WT cC, I66Q and ΔW). RNA-seq analysis was then performed in order to screen for the common DEGs between the WT cC-expressing strain and I66Q-expressing strain or ΔW-expressing strain. For the comparison of WT vs. I66Q with PDI-WT vs. PDI-I66Q, GO analysis indicated the consumption of energy by the host cells as an important factor in the case of foreign protein expression since protein synthesis is an energy-costing process. Glycolysis and the TCA cycle are processes of energy acquisition, so reducing energy acquisition is a way to force the slow-down of protein synthesis because, without ATP and GTP, protein synthesis cannot occur. Glycolysis also leads to the pentose phosphate pathway, which provides nucleotides for nucleic acid synthesis. Thus, lowering glycolysis also affects RNA synthesis, and hence protein synthesis. The result of KEGG pathway analysis showed that in both groups, the common DEGs were enriched in the ribosome and TCA cycle, as well as in carbon metabolism and glycolysis/gluconeogenesis, consistent with the results of GO analysis. The basal metabolic level of *P. pastoris* is closely related to the protein expression level (Liang et al., 2012; Renuse et al., 2014). When *P. pastoris* expressed I66Q and ΔW during the methanol induction stage, the common DEGs enriched in the TCA and glycolytic pathways were significantly decreased compared with those in the WT cC-expressing strain. At the same time, the common DEGs enriched in the protein translation and protein folding processes were up-regulated, indicating an initial increased expression of protein synthesis-related genes, and even glycolysis and the TCA cycle-related genes in the case of the I66Q- and ΔW-expressing strains. At a later time, the burden caused by these misfolded proteins started to affect the cells and resulted in an adjustment to reduce the expression of the foreign proteins. However, the selective pressure of methanol induction was still ongoing, so protein synthesis could only be slowed down by reducing the flow of energy to protein synthesis (Sola et al., 2007; Orman et al., 2009). These findings indicated that when foreign proteins that may not be properly folded are expressed in *P. pastoris*, the cells need to adjust their own metabolic states in order to maintain intracellular homeostasis, based on the degree of protein instability.

Hub genes are considered to be key genes that play vital roles in biological processes and can affect the regulation of other genes in a related pathway (Liu et al., 2022; Shu et al., 2022). It is of significance to note that the key gene *C4QXU7*, a small subunit of the ribosomal protein S28e, was identified by both the comparisons of WT vs. I66Q/ΔW and PDI-WT vs. PDI-I66Q/ΔW (Figure 4B). Yeast ribosomal proteins play important roles in the biogenesis and function of the ribosome (Aw et al., 2017). Deletion of a particular ribosome protein can delay or impair the subunit assembly, indicating that the decelerated elongation stage of translation might promote the co-translational folding rate of heterologous proteins (Liao et al., 2019). In *P. pastoris*, overexpression of xylanase A (a foreign protein) can lead to a significant down-regulation of numerous ribosomal proteins, resulting in decelerated translation elongation and enhanced folding efficiency for xylanase A (Navone et al., 2021b). Meanwhile, studies have shown that knocking out the *C4QXU7* gene in *P. pastoris* does not affect its growth, but can lead to a significant increase in the secretion levels of exogenous proteins (e.g., Pfu and Phytase), indicating that the decelerated elongation rate caused by the loss of C4QXU7 might promote the co-translational folding rate of heterologous proteins, increasing the expression of Pfu and Phytase (Liao et al., 2019). Together with our data on protein expression in *P. pastoris*, these observations could collectively indicate that knocking out the C4QXU7 gene may promote the expression of foreign proteins that are not easily folded in *P. pastoris*.

On the other hand, because *P. pastoris* is widely used for the expression of foreign proteins in industrial protein production, how to design and develop a new *P. pastoris* expression system capable of yielding a high expression level and flexible regulation characteristics is one of the key problems and important goals faced by bioengineering and synthetic biotechnology. In this study, the protein expression levels of three model proteins with the order of decreasing stability WT > I66Q > ΔW were significantly increased in *P. pastoris* GS115 that simultaneously overexpressed PDI. In addition to molecular chaperones, our data also revealed that some ribosomal proteins, e.g., C4QXU7, may also be important targets that can be modulated to increase the expression of foreign proteins. The modulation of key *P. pastoris* ribosomal protein genes will expand its application potential in a broader scenario. From this viewpoint, our research has provided valuable information for developing a convenient platform to improve the efficiency of heterologous protein expression in *P. pastoris*, which may also contribute to the application of synthetic biology in a special field, such as in the field of biofuel production.

## Data availability statement

The data presented in this study are deposited in the BioSample database repository of National Center for Biotechnology Information (NCBI), accession number PRJNA892887.

## Author contributions

JH and JJ: conceived and designed the research. TN, JH, and YC: wrote the manuscript. SQ and XZ: performed the experiments. TN, YC, and NM: performed the data analyses. XS and RW: provided experiment assistance, data curation, and validation. JH and NM: funding acquisition. AC: writing—review and editing. All authors contributed to the article and approved the submitted version.

## Footnotes

1
https://submit.ncbi.nlm.nih.gov/subs/bioproject/SUB12189176/overview
